# Midlife alcohol consumption and later life cognitive impairment: Light drinking is not protective and APOE genotype does not change this relationship

**DOI:** 10.1371/journal.pone.0264575

**Published:** 2022-03-11

**Authors:** E. Julia Chosy, Steven Edland, Lenore Launer, Lon R. White

**Affiliations:** 1 Pacific Health Research and Education Institute, Honolulu, Hawaii, United States of America; 2 University of California at San Diego, La Jolla, California, United States of America; 3 National Institute on Aging, National Institutes of Health, Bethesda, Maryland, United States of America; University of Jyvaskyla, FINLAND

## Abstract

**Introduction:**

Much debate exists about the role of light to moderate alcohol intake and subsequent cognitive function. The apolipoprotein E genotype may modify the relationship.

**Methods:**

Using data from the Honolulu-Asia Aging Study, a longitudinal population-based cohort (n = 2,416), Cox proportional hazards regression analyses were performed to measure midlife alcohol intake (average age = 52 years) and later life cognitive function (average age = 87 years) and to explore the role of apolipoprotein E genotype.

**Results:**

No protective effect of light drinking (>1 drink/month– 1 drink/day) or moderate drinking (>1–2 drinks/day) was observed in the cohort in adjusted models (HR = 1.013, CI:0.88–1.16; HR = 1.104, CI:0.91–1.34, respectively). Heavy drinking (>2–4 drinks/day) and very heavy drinking (>4 drinks/day) increased the risk for incident moderate cognitive impairment (HR = 1.355, CI:1.09–1.68; HR = 1.462, CI:1.04–2.05, respectively). When examining the relationship by apolipoprotein E ε4 carrier status, a similar dose-response pattern was observed in both groups with higher hazard ratios for those carrying at least one copy of the apolipoprotein E ℇ4 allele. As alcohol level increased, the age at incident moderate cognitive impairment decreased, especially among those with at least one apolipoprotein E ℇ4 allele.

**Discussion:**

We did not observe a significant protective effect for light to moderate drinking in midlife and subsequent cognitive impairment in this cohort. Heavy drinking increased the risk for moderate cognitive impairment and decreased the age at incidence, as did carrying at least one allele of the apolipoprotein E ℇ4 gene.

## Introduction

Little consensus exists as to whether there is a level of alcohol consumption that is beneficial or protective for cognitive function. Results from studies examining alcohol consumption and later life cognitive function have been varied and unpredictable, with meta-analyses and reviews showing differing results [1–8]. While evidence supports a deleterious influence of heavy alcohol consumption on cognition [9–14], the role of moderate alcohol intake has been debated. Several studies have shown a decrease in risk of cognitive impairment with light to moderate alcohol consumption, reflected by a J-shaped curve describing the relationship [15–21]. This has been shown both for drinking patterns in middle age [22–24] and later life [25–27]. Other studies have shown no effect of light to moderate alcohol consumption on cognitive function [9, 18, 28–30]. Topiwala et al. [31] found that all alcohol consumption, even at moderate levels, is associated with adverse brain outcomes, and Zhou et al. [32] showed that daily drinking among older men was associated with an increased risk for both Alzheimer’s disease (AD) and vascular dementia.

An additional issue is the role of the apolipoprotein E (APOE) genotype. APOE plays a role in the transport and redistribution of lipids. Of the three common isoforms of this glycoprotein (ℇ2, ℇ3, ℇ4), ℇ4 has been associated with a higher risk of dementia and cognitive decline [33]. Some studies have shown an effect of alcohol consumption on cognition that is dependent on the presence or absence of the APOE ℇ4 allele [5, 22, 34, 35]. Dufouil et al. [36] found that drinking was associated with a decreased risk of cognitive deterioration, but only among those without an APOE ℇ4 allele. The opposite association was seen among individuals with at least one copy of the allele. Luchsinger [37] found that consumption of up to three servings of wine daily is associated with lower risk of AD in older individuals without the APOE ℇ4 allele, but not among those with the allele. Still others have found no effect of the presence or absence of the APOE ℇ4 allele on alcohol use and cognitive function [38–40].

Using data from the Honolulu-Asia Aging Study (HAAS), a population-based, longitudinal cohort of Japanese-American men, we examined relationships between midlife alcohol consumption and risk of cognitive impairment in later life. A previous study in this cohort found a positive association for moderate alcohol intake and poorer cognitive performance 18 years later [41]. The current study takes advantage of an additional 20 years of follow-up data and examines the relationship using survival analysis. Additionally, we examine the effect of the APOE genotype on the relationship between alcohol intake and cognition. The current study adds uniquely to the literature due to its extensive follow-up time (nearly 40 years) and its investigation of the role of the APOE genotype in the relationship between alcohol consumption and subsequent cognitive decline.

## Methods

### Study population

Study participants came from HAAS, which evolved from the Honolulu Heart Program (HHP), both of which were reviewed and approved by the Institutional Review Board of Kuakini Medical Center, Honolulu, Hawaii. Beginning in 1965, a large, representative HHP cohort (n = 8,006) of Japanese men born from 1900–1919 and living on the island of Oahu, Hawaii, were followed and examined at three points over time between 1965 and 1975. Starting in 1991, approximately 80% of the surviving cohort (n = 3,734) participated in additional follow-up assessments designed to study cognitive function and diseases of aging, which formed the HAAS cohort [42]. The evaluations included data collection on cognitive function, health status, and lifestyle behaviors.

For this study, data from HHP exam 1 (1965–1968) and exam 3 (1971–1975) were used for midlife measures, including alcohol consumption. Data from HAAS exams 4-12 (1991-2012) were used for cognitive measures. Respondents were excluded if already had cognitive impairment at baseline (n = 587), if they were missing data for apolipoprotein E ε4 status (n = 74), alcohol intake (n = 159), or if they had data only for the first HAAS exam (exam 4; n = 508) as they added no time to the survival analysis. The remaining sample comprises 2,416 respondents with an average age at HHP exam 1 of 51.7 years (SD = 4.0), an average age at HAAS exam 4 of 77.1 years (SD = 3.9), and an average age at final cognitive assessment of 86.8 (SD = 5.5). The average length of time between baseline and death was 37.6 years (SD = 5.0).

### Definition of cognitive impairment

The Cognitive Abilities Screening Instrument (CASI) is a comprehensive screening instrument specifically designed for the cross-cultural assessment of cognitive impairment and dementia [43]. It is a composite of the Hasegawa Dementia Screening Scale, the Mini-Mental State Examination, and the Modified Mini-Mental State Examination that combines measures of attention, concentration, orientation, memory, language, visual construction, abstraction, and judgement to create a total score ranging from 0–100. For this analysis, a CASI score of 74 or greater was taken to indicate negligible or no cognitive impairment, while a score of 60–73.9 was considered to reflect moderate cognitive impairment and a score of 0–60 was considered to denote severe cognitive impairment. A score of 23 on the Mini-Mental State Examination, a common indicator of cognitive impairment, corresponds approximately to a CASI score of 74. The HAAS sample was restricted to respondents whose baseline (HAAS exam 4) CASI score was 74 or greater in order to start with a cognitively unimpaired sample. More than 90% of the included participants had died before the final examination cycle. The last recorded CASI score available for each respondent was used to determine the presence (n = 1,009) or absence (n = 1,329) of moderate cognitive impairment during the full course of their participation. Median final exam for those with moderate cognitive impairment was exam 8, while it was exam 7 for those without moderate cognitive impairment. Age at incident CASI score below 74 was calculated by interpolating between follow-up exams assuming a linear decline between subsequent exams. Once a participant fell below 74, they rarely tested above the threshold in future exams. Age at death was 90.3 years for those with moderate cognitive impairment and 87.6 years for those without.

### Definition of alcohol consumption

At both HHP exams 1 and 3 respondents were asked about their quantity and frequency of consumption of beer, wine, liquor, and sake. Ounces of ethanol per month were calculated based on typical alcohol contents at the time in the following manner: grams of ethanol per month = [(number of bottles/cans of beer per month) * (12 oz per beer) * (0.037 oz ethanol/oz of beer)] + [(number of glasses of wine per month) * (4 oz per glass) * (0.1 oz ethanol/oz of wine)] + [(number of liquor drinks per month) * (1.5 oz per drink) * (0.38 oz ethanol/oz liquor)] + [(number of sake drinks per month) * (6 oz per drink) * (0.16 oz ethanol/oz sake)]. All forms of alcohol were combined to produce a robust sample size. To convert ounces per month to drinks, we used 0.6 oz of ethanol to equal one drink, aligning with the definition given by the National Institute on Alcohol Abuse and Alcoholism (NIAAA). In order to acquire a measure of alcohol consumption that would be more representative of intake over time, we averaged the drinks per month from both midlife HHP exams. This was then converted into a 5-level measure of alcohol consumption: non-drinkers (≤1 drink/month), light drinkers (>1 drink/month to 1 drink per day), moderate drinkers (>1–2 drinks/day), heavy drinkers (>2–4 drinks/day), and very heavy drinkers (>4 drinks/day). For analyses examining APOE genotype, heavy and very heavy drinkers were collapsed into a single category due to sample size limitations.

### Covariates

Data were collected on a range of health status measures and lifestyle behaviors at all exams. Laboratory measures included systolic blood pressure (SBP), total blood cholesterol, height, and weight (for body mass index (BMI) calculation). Self-reported health measures at the baseline HAAS exam included history of coronary heart disease, diabetes, and demographic variables (age, educational attainment, marital status, home ownership, and smoking status). Stroke was measured by self-report and medical record review. APOE genotyping was performed by PCR amplification followed by restriction enzyme digest as described in detail previously [44]. Heterozygotes (n = 449, 18.6%) and homozygotes (n = 5, 0.21%) for the APOE ℇ4 allele were combined and designated as the APOE ℇ4 category.

### Statistical analyses

For comparisons across levels of alcohol consumption, Pearson chi-square and ANOVA tests were performed based on the variable classification. Tukey’s post-hoc tests were used to identify differing categories. Multivariate Cox proportional hazards regression models were used to estimate hazard ratios (HR) and 95% confidence intervals (CI) for cognitive impairment by alcohol consumption. Age was used as the time-scale [45] and the cohort was left-truncated by age at baseline HAAS exam. The proportional hazards assumption, assessed by plotting Schoenfeld residuals against time, was met. The referent group was nondrinkers, and the fully adjusted models controlled for education, APOE ℇ4 carrier status, average SBP over HHP exams 1–3, average BMI over exams 1 and 3, history of stroke at the HAAS exam 4, and smoking status at exam 1. Sensitivity analyses were completed among a subset of respondents with no history of heart attack or stroke by exam 4. Statistical significance was defined an as alpha of less than 0.05. All analyses were conducted using SAS 9.4 (Cary, NC).

## Results

Table 1 displays demographic and health status variables across levels of alcohol intake from exams 1–3. At midlife, 31% of respondents reported no or very little (≤1 drink/month) alcohol intake. The majority of respondents (44%) reported light drinking (>1 drink/month- 1 drink/day), while 13% reported moderate (>1–2 drinks/day), 9% reported heavy (>2–4 drinks/day), and 3% reported very heavy (>4 drinks/day) alcohol consumption. Non-drinkers were older at baseline than light drinkers. Non-drinkers died at an older age than heavy and very heavy drinkers. Non-drinkers and light drinkers had a lower average SBP than moderate and heavy drinkers, while very heavy drinkers had the highest proportion of current smokers. Among those who ultimately developed moderate or severe cognitive impairment, very heavy drinkers had an age at onset approximately 2.1 years earlier than non-drinkers.

**Table 1:** Comparison of HAAS characteristics across level of alcohol consumption in midlife.

Parameter	Midlife Alcohol Intake (average of exams 1 and 3)					*P* value^a^
	Non-Drinker (≤1 drink/month)	Light (>1 drink/month–1 drink/day)	Moderate (>1–2 drinks/day)	Heavy (>2–4 drinks/day)	Very Heavy (>4 drinks/day)	
	% (#), Mean (SD)					
**TOTALS**	30.9% (747)	44.1% (1066)	12.5% (302)	9.2% (223)	3.2% (78)	
**Demographics**						
Age at E1	52.0 (4.2)	51.4 (3.9)	51.6 (3.8)	51.5 (4.1)	51.7 (3.9)	0.0381*
Age at death	89.3 (5.2)	89.0 (5.1)	88.5 (5.1)	88.0 (5.0)	87.2 (5.2)	0.0004***
Years of education	11.0 (3.2)	11.2 (3.1)	10.7 (3.2)	10.4 (2.9)	9.9 (3.1)	< .0001***
Ever married	97.6% (729)	98.3% (1048)	98.0% (296)	99.1% (221)	97.4% (76)	0.6107
Own home	80.2% (599)	83.5% (889)	78.8% (238)	79.8% (178)	74.4% (58)	0.0945
APOE ε4	18.2% (136)	18.3% (195)	20.9% (63)	18.8% (42)	23.1% (18)	0.7122
**Midlife Health Status (E1-3)**						
Average BMI, E1,3	23.9 (2.8)	24.0 (2.7)	24.1 (2.8)	24.0 (2.8)	24.0 (2.9)	0.8858
Average SBP, E1-3	128.3 (15.2)	129.3 (15.6)	131.9 (15.8)	135.4 (16.6)	132.7 (16.1)	< .0001***
Average cholesterol, E1-3	217.8 (35.3)	212.4 (29.0)	217.6 (28.3)	209.5 (26.5)	210.3 (29.5)	0.1924
Current smoker, E1	26.1% (195)	34.4% (367)	38.4% (116)	48.0% (107)	65.4% (51)	< .0001***
**Later Life Health Status (E4)**						
Heart attack, ever	7.4% (54)	6.8% (72)	6.0% (18)	6.3% (14)	5.1% (4)	0.9022
Coronary heart disease	29.2% (218)	27.7% (295)	25.5% (77)	22.4% (50)	30.8% (24)	0.2883
Stroke, ever	6.6% (49)	7.8% (83)	8.6% (26)	10.3% (23)	9.0% (7)	0.4073
Diabetes	18.2% (134)	16.4% (173)	12.8% (38)	14.5% (32)	18.0% (14)	0.2542
Cancer, ever	9.3% (69)	8.1% (85)	8.9% (27)	7.8% (17)	9.0% (7)	0.8882
CES-D score >9	14.5% (98)	14.1% (136)	17.2% (47)	16.3% (32)	16.4% (11)	0.2895
**Cognitive Function**						
Baseline CASI, E4	88.0 (6.1)	88.6 (6.0)	88.2 (5.9)	86.9 (6.3)	86.5 (6.4)	0.0006***
Final CASI	70.3 (21.3)	71.1 (21.1)	70.6 (22.3)	69.1 (22.2)	70.8 (17.1)	0.7864
Incident CASI <74	47.4% (354)	46.6% (497)	48.0% (145)	51.1% (114)	50.0% (39)	0.7854
**Among those with CASI <74**						
Average age at incident	84.7 (5.1)	84.4 (5.1)	83.7 (5.6)	83.2 (4.8)	82.6 (4.9)	0.0095**
Final CASI	55.1 (21.8)	55.4 (21.2)	55.2 (23.2)	54.3 (21.9)	58.8 (15.8)	0.9482

Note. Dichotomous measures are shown as percentage and sample size while continuous measures are displayed as mean and standard deviation (SD).

Abbreviations: APOE ε4, apolipoprotein E ε4 allele; BMI, body mass index; CASI, Cognitive Abilities Screening Instrument; CES-D, Center for Epidemiologic Studies-Depression scale; E, exam; SBP, systolic blood pressure; SD, standard deviation.

^a^p value for Pearson chi-square or F test.

*p < .05

**p < .01

***p < .001.

The crude and adjusted HRs estimating the impact of midlife drinking on later life cognitive function are shown in Table 2. Light and moderate midlife drinkers had no difference in risk of moderate or severe cognitive impairment compared to non-drinkers in both crude and adjusted models. However, heavy drinkers at midlife had a 36% increase in risk compared to non-drinkers (HR = 1.355, CI = 1.09–1.68) and very heavy drinkers had a 46% increase in risk compared to non-drinkers (HR = 1.462, CI = 1.04–2.05) after adjusting for covariates. Running the analysis among respondents with no history of heart attack or stroke at exam 4 resulted in the same observed pattern.

**Table 2:** Hazard ratios and confidence intervals for moderate cognitive impairment across levels of alcohol intake at midlife.

Moderate Cognitive Impairment	Midlife Alcohol Intake (average of exams 1 and 3)				
	Non-drinker (≤1 drink/month)	Light (>1 drink/month–1 drink/day)	Moderate (>1–2 drinks/day)	Heavy (>2–4 drinks/day)	Very heavy (>4 drinks/day)
	Hazard Ratio (95% Confidence Intervals)				
Crude	1	0.963 (0.84–1.10) p = 0.5843	1.088 (0.90–1.32) p = 0.3933	**1.515 (1.23–1.88) p = 0.0001**	**1.700 (1.22–2.37) p = 0.0017**
	n = 747	n = 1,066	n = 302	**n = 223**	**n = 78**
Adjusted^a^	1	1.013 (0.88–1.16) p = 0.8515	1.104 (0.91–1.34) p = 0.3217	**1.355 (1.09–1.68) p = 0.0060**	**1.462 (1.04–2.05) p = 0.0270**
	n = 743	n = 1,062	n = 302	**n = 223**	**n = 78**

Note. Bolding indicates significant results at p < .05; Abbreviations: E, exam.

^a^Adjusted for education, apolipoprotein E ε4 allele status, average body mass index (E1,3), average systolic blood pressure (E1-3), history of stroke (E4), and smoking status (E1).

Although the interaction term between alcohol consumption and APOE ℇ4 carrier status was not significant, we stratified the results to more closely examine this relationship. The HRs for level of alcohol intake at midlife, APOE ℇ4 carrier status, and later life cognitive impairment (moderate or severe) are shown in Table 3. Among those who do not carry any APOE ℇ4 alleles, a similar relationship is seen as above. Light and moderate drinkers have no difference in risk of moderate or severe cognitive impairment in life (HR = 1.042, CI = 0.89–1.22; HR = 1.009, CI = 0.81–1.26, respectively) while heavy-to-very-heavy drinkers show a 47% increased risk as compared to non-drinkers (HR = 1.473, CI = 1.18–1.83). Compared to respondents without an APOE ℇ4 allele, those with at least one copy showed an increased risk of cognitive impairment at all levels of midlife alcohol consumption. However, the risk grows with growing alcohol intake, reflecting a similar dose-dependent relationship observed among those without the APOE ℇ4 allele. Non-drinkers with at least one APOE ℇ4 allele demonstrated a 35% increased risk of later life moderate cognitive impairment as compared to non-drinkers with no copies of the ℇ4 allele (HR = 1.352, CI = 1.04-1.75). The risk for cognitive impairment increases with alcohol consumption as seen in moderate (HR = 2.039, CI = 1.45–2.87) and heavy-to-very-heavy (HR = 1.544, CI = 1.07–2.23) drinkers. Although the HR is greater for moderate drinkers than heavy-to-very-heavy drinkers, it is important to note the confidence intervals overlap, suggesting no statistical difference between the HR estimates.

**Table 3:** Hazard ratios and confidence intervals for moderate cognitive impairment across levels of alcohol intake at midlife by APOE ε4 status.

	Midlife Alcohol Intake (average of exams 1 and 3)			
Moderate Cognitive Impairment	Non-drinker (≤1 drink/month)	Light (>1 drink/month–1 drink/day)	Moderate (>1–2 drinks/day)	Heavy/Very Heavy(>2 drinks/day)
	Hazard Ratio^a^ (95% Confidence Intervals)			
APOE ε4 Negative	1	1.042 (0.89–1.22) 0.5492	1.009 (0.81–1.26) p = 0.9431	**1.473 (1.18–1.83) p = 0.0006**
	n = 608	n = 868	n = 239	**n = 241**
APOE ε4 Positive	**1.352 (1.04–1.75) p = 0.0209**	1.196 (0.95–1.51) p = 0.1095	**2.039 (1.45–2.87) p = < .0001**	**1.544 (1.07–2.23) p = 0.0260**
	**n = 135**	n = 194	**n = 63**	**n = 60**

Note. Bolding indicates significant results at p < .05; Abbreviations: APOE ε4 apolipoprotein E ε4 allele; E, exam.

^a^Hazard ratios are adjusted for education, average body mass index (E1,3), average systolic blood pressure (E1-3), history of stroke (E4), and smoking status (E1).

**Fig 1:**
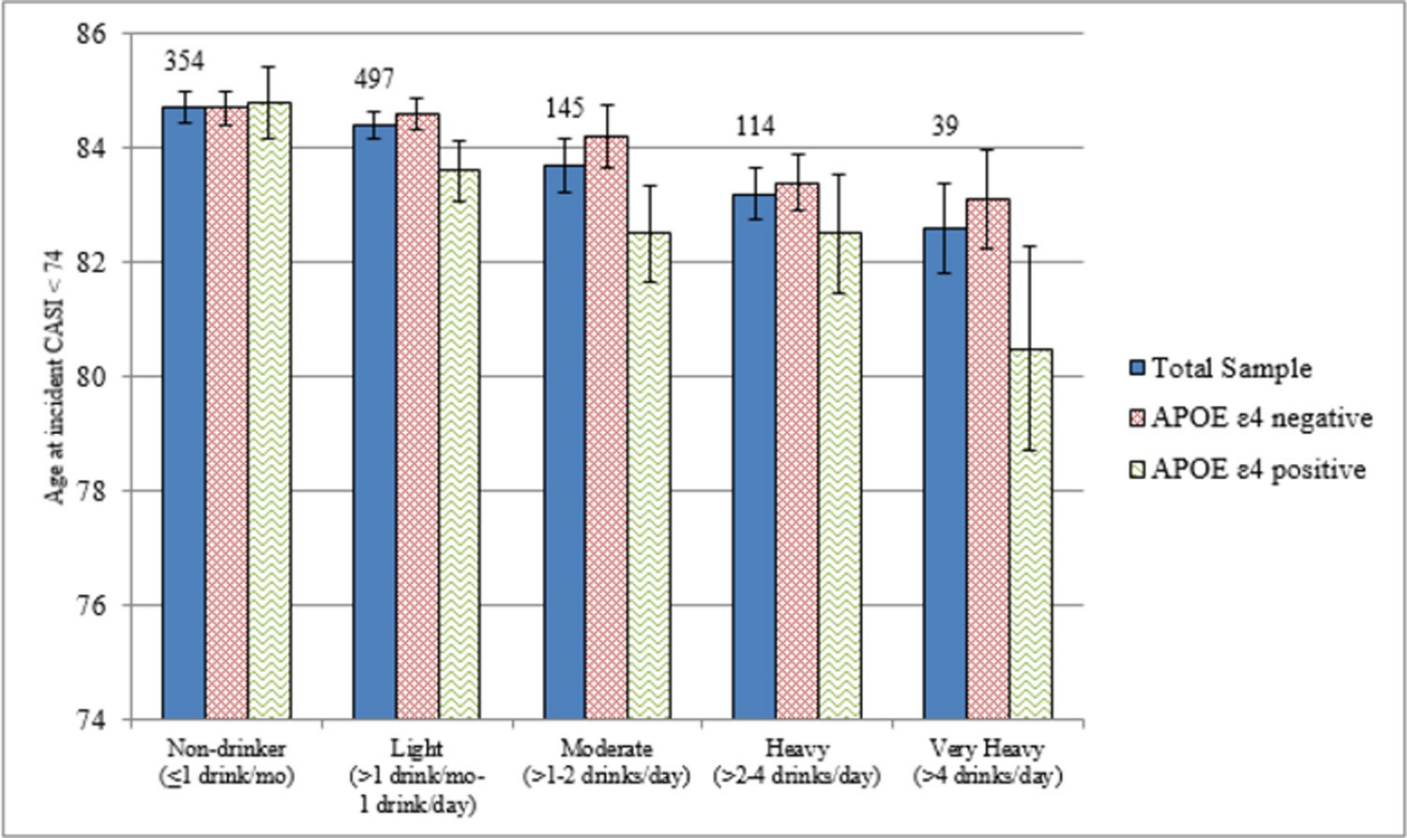
Age of onset of moderate cognitive impairment by midlife alcohol intake (exams 1,3) and apolipoprotein E ℇ4 status. Bars represent standard errors of the estimates and numbers above the bars reflect sample size for the total sample.

Fig 1. shows the age at which the participants’ CASI test score is estimated to have fallen below 74, indicating the onset of decline or age of incident moderate cognitive impairment. Among the decedents who developed moderate cognitive impairment, the age at onset appears to decrease with increasing alcohol consumption and with APOE ℇ4 status. Nondrinkers have approximately the same age at onset regardless of APOE ℇ4 status. The age at onset decreases as alcohol consumption increases but appears to decrease faster among respondents with the APOE ℇ4 allele. Although the difference by APOE ℇ4 status for light alcohol intake was marginally significant (p = 0.08), there were no statistically significant differences between the ages at onset. The small sample size may not lend enough strength to observe statistical differences.

## Discussion

The results from the current all-male cohort do not support the suggestion that moderate midlife drinking is beneficial or protective for cognitive function later in life. Discrepant findings in the literature may be due to differences in alcohol classification, study design, follow-up time, and bias. In a recent systematic review of 27 cohort studies, Brennan et al. [2] found only slightly better cognition in female moderate drinkers and a trivial effect in males. They warn that differences among the included studies made it impossible to discern if the effect was true or due to bias. Another issue is selection of the reference group. Abstainers may include individuals who quit because of health reasons and may bias the reference group toward higher levels of ill health. Several health conditions have been associated with dementia in this cohort, including diabetes [46] and hypertension [47], which may increase the prevalence of dementia in a group with ill health. For better distribution, we included respondents with a very low level of alcohol intake (≤1 drink/month) in our ‘non-drinker’ category. This may have diluted out the ‘sick quitters’ and led to a more representative comparison group.

We found an increased risk for cognitive impairment among the heavy (>2–4 drinks/day) and very heavy (>4 drinks/day) drinkers, a result supported by many studies [8–10, 19, 32]. As ethanol is a known neurotoxin, this is not a surprising finding, but still an important one. Current public health messaging suggests moderate drinking (≤2 drinks per day for men, ≤1 drink per day for women) is safe. In our study, just one extra drink a day (among men) resulted in increased risk of cognitive impairment. Among respondents with the APOE ℇ4 allele, even moderate drinking showed an increased risk for subsequent cognitive impairment. Thus, the line demarking safe drinking is thin and varies with genetic factors. These issues need to be taken into consideration when dispensing health advice on alcohol consumption.

The presence of at least one APOE ℇ4 allele increased the risk for moderate cognitive impairment in an approximately additive manner. That is, a similar relationship between alcohol and cognition was observed among those with the APOE ℇ4, only the risk was shifted higher (Table 3). This finding is supported by several other studies [38–40, 48]. In a review, Panza et al. [5] postulates that the protective effect of alcohol against cognitive impairment may be more likely in the absence of the APOE ℇ4 allele. While we did not observe a protective effect among non-carriers, there was a slight decrease in risk among light drinkers who carry the APOE ℇ4 allele. However, it was not strong enough to indicate protection from cognitive impairment. The risk of cognitive impairment among those with the APOE ℇ4 allele was highest among the moderate and heavy drinkers. Though the HR was higher among moderate drinkers than heavy-to-very-heavy drinkers, this may be due to the small sample size (the CI’s overlap for these two measures) or to a hardy survival effect in the heavy-to-very-heavy drinkers.

Harwood et al. [49] found that a history of heavy drinking (> 2 drinks per day) and the presence of an APOE ℇ4 allele both reduced the age of onset of AD by 2–3 years. We also saw a reduction in the age at onset of moderate cognitive impairment based on heavy alcohol consumption (>2 drinks/day) and presence APOE ℇ4 (Fig 1). Though the differences were not significant, there is an observed 4.3-year discrepancy for age at onset of cognitive impairment when comparing APOE ℇ4 carriers who drink heavily to non-drinkers who do not carry the allele.

As with other research of similar design, there are limitations to be considered when interpreting the results of this study. Any information collected via self-report is subject to recall bias that may affect the accuracy of the results. Particularly alcohol intake has been observed to be underestimated in self-reports [50]. The composition of the sample brings generalizability of the results into question, given the cohort consists only of men with Japanese ancestry. However, previous studies using the HAAS data have shown similar findings as other cohorts, including the Nun Study, the Rush Religious Orders Study, and the Rush Memory and Aging Project [42, 51, 52].

Some may argue that measuring alcohol at only two points in midlife does not accurately reflect alcohol consumption over the lifespan. While this may be true, we observed a great deal of correlation between the two points in midlife (r = 0.521, p < .0001) and between the later point in midlife and the measurement taken in later life (r = 0.455, p < .0001). This suggests drinking habits remained relatively constant over time in this cohort. This does not necessarily reflect consumption as young adults, but we did not have access to those data.

The current study has many strengths, including the longitudinal study design, large cohort size, and high participation rates. Data were collected prospectively, reducing bias, and there was a long follow-up period for monitoring cognition, up to 48 years. Lastly, collecting data on alcohol intake at two time points allowed us to create a measure of alcohol that better reflected consumption over midlife rather than at a single time point.

## Conclusion

We did not observe a protective effect on later life cognitive function for light to moderate consumption of alcohol in midlife. Heavy alcohol consumption increased the risk for cognitive impairment later in life. The presence of at least one APOE ℇ4 allele increased the risk for moderate cognitive impairment with increasing alcohol consumption. As alcohol level increased, the age at incident moderate cognitive impairment decreased, especially among those with at least one APOE ℇ4 allele.
